# Supplementary material for: Measuring gas discharge in contact electrification
Source: Nat Commun. 2023 Dec 7;14:8100. doi: 10.1038/s41467-023-43721-1 (PMC10703932; doi:10.1038/s41467-023-43721-1)
Supplement: Supplementary file 1 — Supplementary Information [file 41467_2023_43721_MOESM1_ESM.pdf]

Supplementary Information

Measuring gas discharge in contact electrification

Hongcheng Tao[1], James Gibert[1]*
[1]School of Mechanical Engineering, Purdue University, West Lafayette, IN, USA. *Email: jgibert@purdue.edu

Supplementary Method 1

Preliminary results of a supplementary test conducted in room air provide evidence for the accuracy of surface charge density estimation via Coulomb force measurement while determining the polarity of contact electrification, which is difficult in the vacuum chamber. Two sample surfaces are first brought into controlled contact cycles and then transferred onto a low-capacity load cell where the Coulomb force is measured at a fixed target gap distance, as shown in Supplementary Fig. 1. A sharp-tip electrode (brush) grounded through an electrometer (Keithley 6514) is then swept over each surface successively so that the majority of the surface charge is collected via tip breakdown by the brush and measured by the electrometer. Supplementary Fig. 1c depicts the time histories of load cell and electrometer readings for a typical test run using a PDMS-PTFE contact pair with a 35-mm-diameter circular effective contact area. The samples undergo 5 contact cycles with a peak force of 4 N (4.16 kPa) and then separate to a gap around 50 mm to induce adequate air breakdown discharge so that the surface charge remains quasi-constant in later steps when the sample is transferred to the low-capacity load cell and the gap is closed. The surfaces are brought to a target gap distance of 1.5 mm where a 6 mN Coulomb force is measured to give an estimated surface charge density of 11.70 μC/m², while direct surface charge collection by the brush electrode reads 10.7 nC for the PDMS surface and −11 nC for the PTFE surface, yielding surface charge densities of 11.12 μC/m² and −11.43 μC/m², respectively.

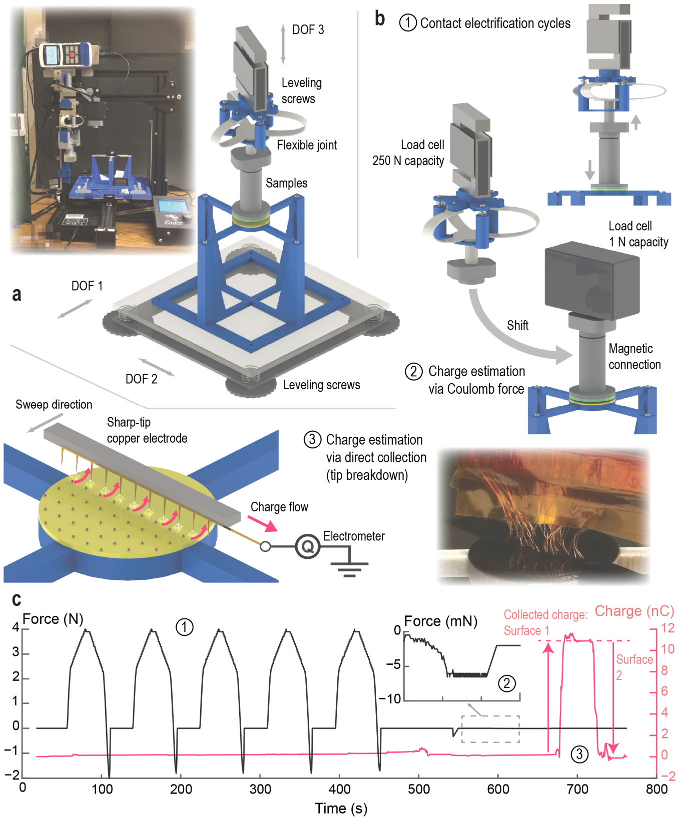

Supplementary Fig. 1 | Preliminary tests in room air. a Test apparatus. **b** Test steps include 1) contact electrification cycles, 2) Coulomb force measurement and 3) surface charge collection using a copper brush grounded through an electrometer. **c** A typical test with time histories of load cell and electrometer readings.

Supplementary Discussion 1

Supplementary Fig. 2 describes theoretical mechanisms of secondary electron emission from the negatively charged surface to clarify discussions in the main text regarding variations in the estimated secondary-electron-emission coefficients shown in Fig. 3d.

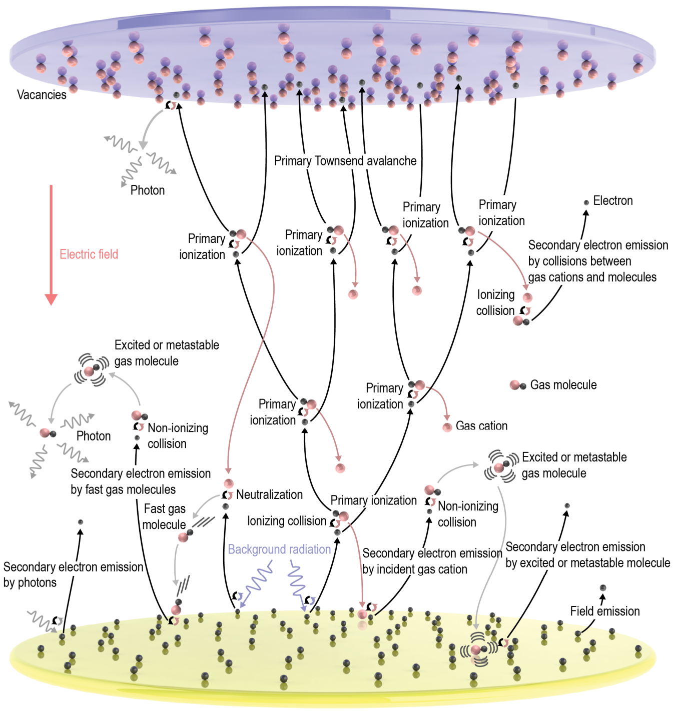

Supplementary Fig. 2 | Theoretical mechanisms of secondary electron emission from the negatively charged surface that contribute to sustaining Townsend avalanches in the gas breakdown during contact electrification. Including but not limited to: that by gas cations, excited or metastable gas molecules as well as photons emitted from them, fast gas molecules created by neutralization of accelerated cations near the negatively charged surface, and photons released from the positively charged surface by incident electrons, along with potential secondary electron emission from ionizing collisions between gas cations and molecules. Primary electron emission mechanisms include background radiation and potential field emission.

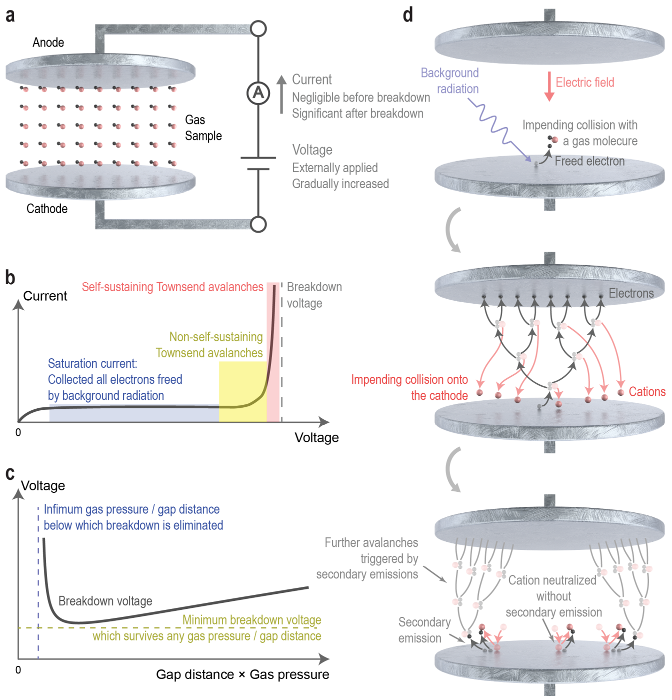

Supplementary Fig. 3 | Gas breakdown between electrodes. a Test setup with electrodes submerged in the examined gas, connected to a voltage source with continuous charge supply. **b** Typical relation between the current across the gap and the voltage applied, for a fixed combination of gap distance and gas pressure, with definition of the corresponding breakdown voltage. **c** General shape of Paschen's law (the Paschen curve) for a fixed combination of gas type and electrode materials, showing the dependence of breakdown voltage on the product of gap distance and gas pressure. **d** Mechanism of gas breakdown explained as self-sustaining cascades of Townsend avalanches.

Supplementary Discussion 2

Supplementary Fig. 3 displays the classical Townsend's theory for gas breakdown between electrodes with an applied voltage to clarify corresponding discussions in the main text.

Supplementary Discussion 3

The presented test setup is applied to perform PDMS-iron electrification with a small effective contact area to achieve an apparent charge saturation for comparison with surface charge densities characterized in literature, as shown in Supplementary Fig. 4. The same PDMS sample as in the presented PDMS-acrylic tests is used, while the iron sample is circular with a 24.3 mm diameter (4.62 cm^2, commercially available, Prince Frederick Store), sanded to 5000 grits and cleaned with isopropyl alcohol. After 50 contact cycles under 20 N peak force in 10 Pa vacuum a surface charge density of 480 μC/m^2 is measured, which is higher than, but comparable in magnitude to, a reported value of 243 μC/m^2 in literature[1].

Supplementary Discussion 4

Details of the test setup, PDMS sample fabrication and surface alignment/leveling are shown in Supplementary Fig. 5, while Supplementary Fig. 6 explains characterization of the load cell linear deflection stiffness. Meanwhile, in Supplementary Movie 1 the load cell oscillations at sudden dissipation of van der Waals force (surface separation) and Coulomb force (gas breakdown events) are displayed using the PDMS-iron setup.

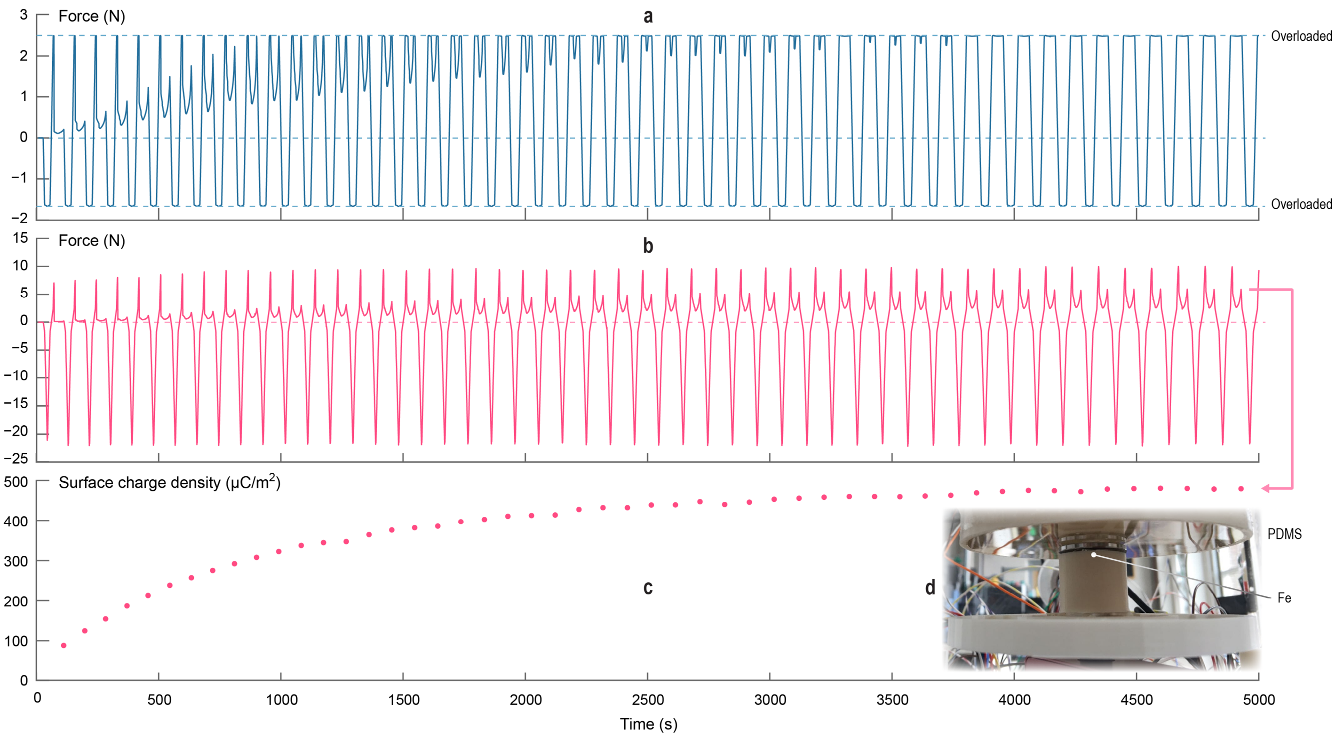

Supplementary Fig. 4 | PDMS-iron contact electrification cycles. a Time history of bottom load cell readings, positive being attraction. **b** Time history of top load cell readings, positive being attraction. **c** Surface charge density estimated from top load cell readings. **d** Sample surfaces.

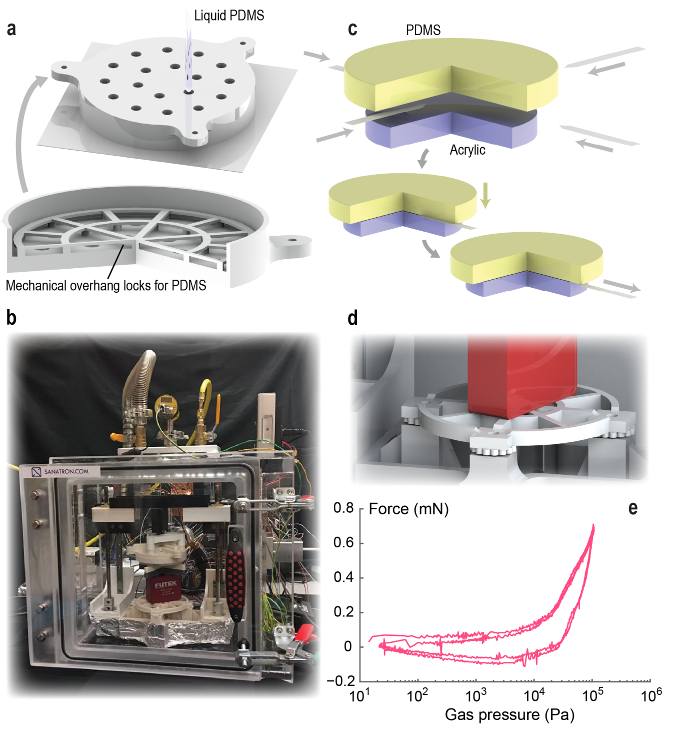

Supplementary Fig. 5 | Test setup and configuration. a Structure of the ASA plastic mold for the PDMS sample showing overhang locks to be buried in cured PDMS. **b** Picture of the test apparatus excluding the gas cylinder, vacuum pump, load cell amplifiers and data acquisition terminal. **c** Calibration of surface alignment using a paper strip. **d** Leveling screws connecting the bottom load cell and the base. **e** Drift of bottom load cell reading with respect to gas pressure.

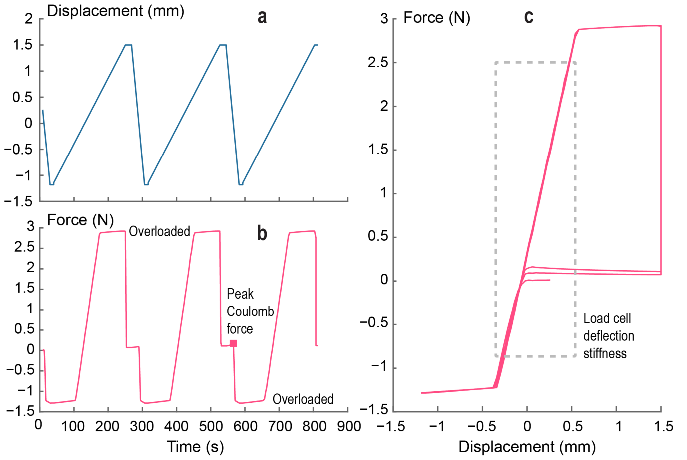

Supplementary Fig. 6 | Load cell deflection. a Displacement time history of the top sample-load cell assembly in typical contact cycles. **b** Time history of the bottom load cell readings. **c** Estimation of load cell deflection stiffness from the linear operational region of the bottom load cell.

Supplementary Discussion 5

Supplementary Fig. 7 compares the van del Waals adhesions observed in PDMS-acrylic (strong adhesion) and copper-nylon (trivial adhesion) tests by showing time histories of the top load cell readings during typical charging cycles. The PDMS-acrylic contacts are performed at a gas pressure around 6 Pa and, as a conservative reference, the Coulomb-force–displacement relation in the pseudo-constant-pressure test run at around 13 Pa is plotted in Supplementary Fig. 7b, showing first discharge events at approximately 2.7 mm and 3.9 mm, which are significantly greater than the maximum gap distance of 1.5 mm in the contact cycles to prevent undesired charge dissipation by gas breakdown.

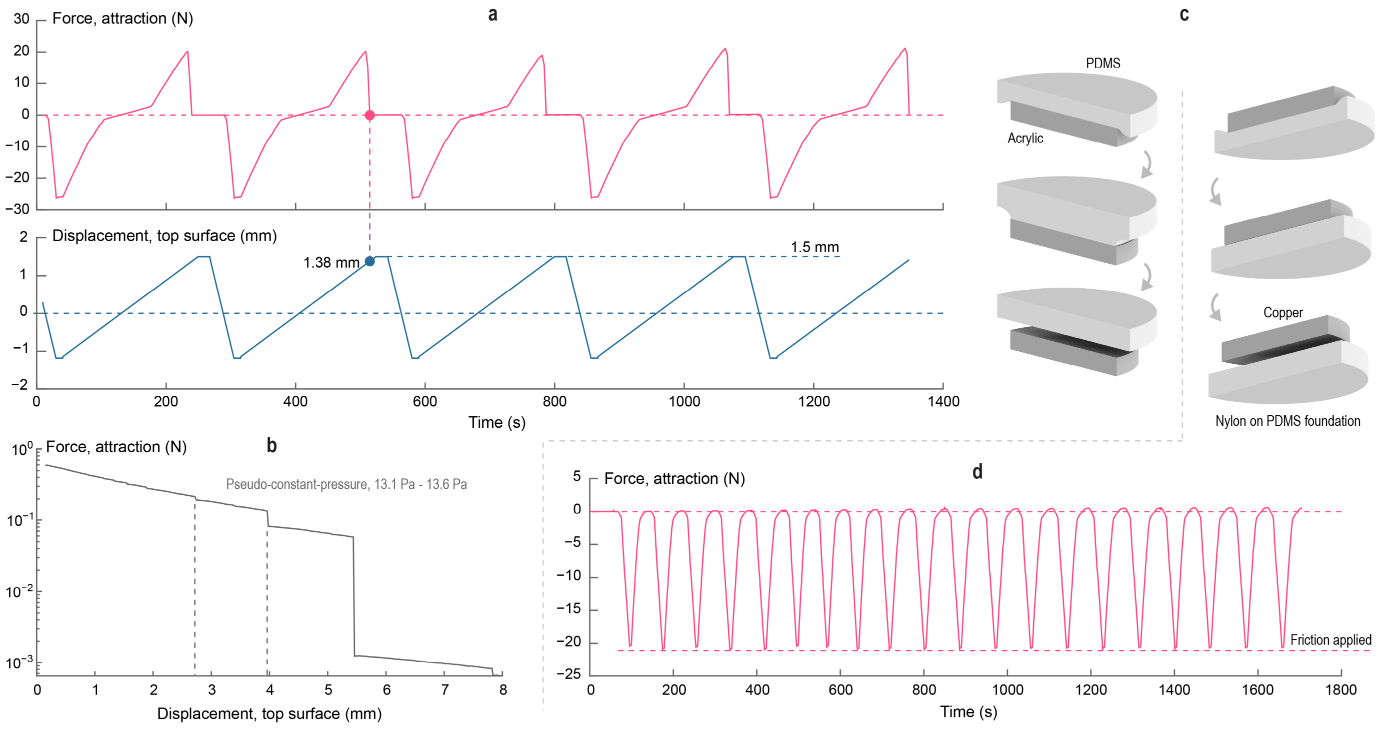

Supplementary Fig. 7 | Details of charging cycles. a Time histories of top load cell readings and top surface displacements in typical PDMS-acrylic charging cycles in 6 Pa nitrogen. **b** Coulomb force measurement in a pseudo-constant-pressure PDMS-acrylic test for nitrogen breakdown at around 13 Pa. **c** Schematics of differences in the magnitude of van del Waals adhesion in the separation phase of PDMS-acrylic and copper-nylon contact cycles. **d** Time history of top load cell readings for typical copper-nylon charging cycles.

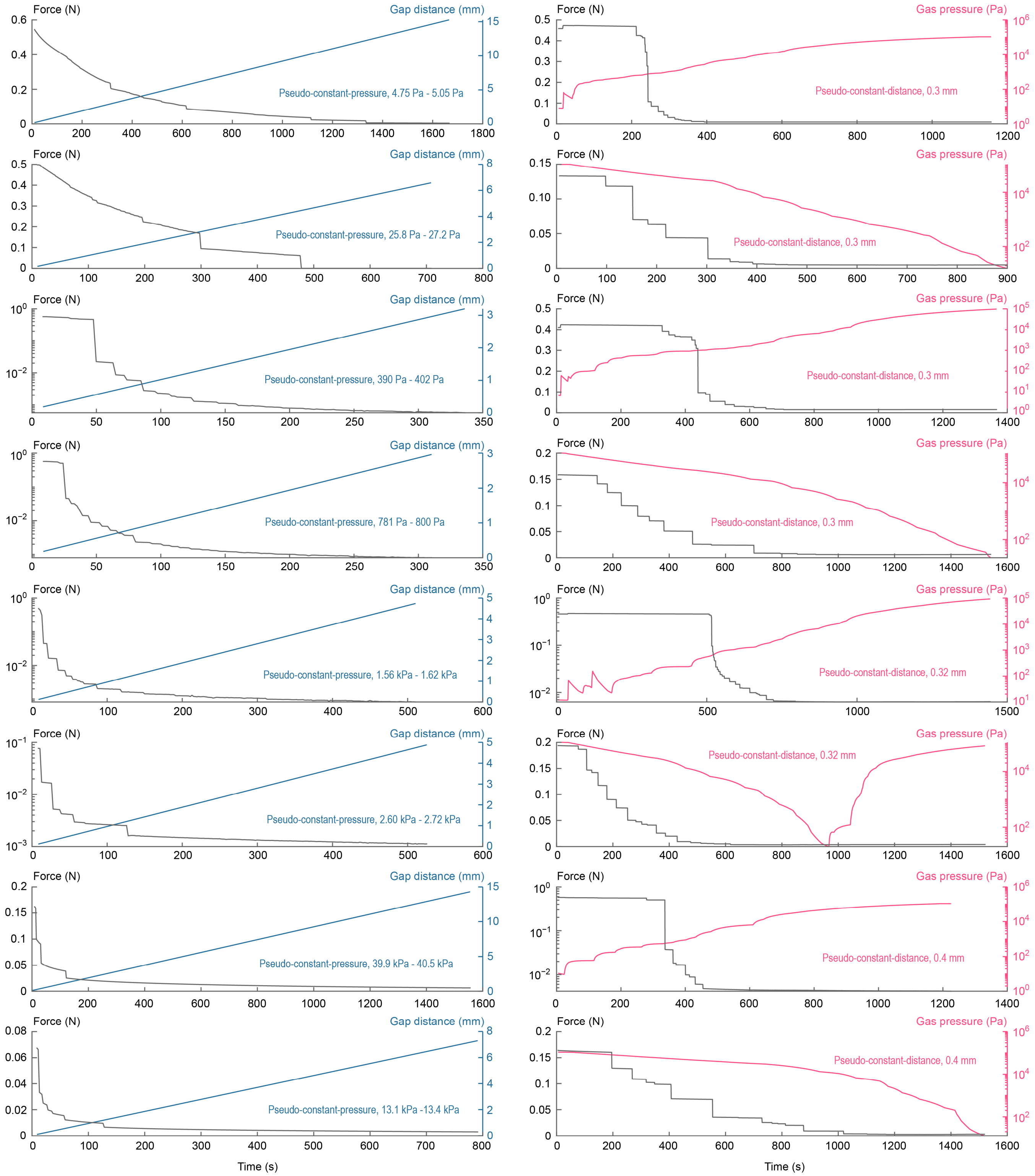

Supplementary Fig. 8 | Time histories of Coulomb force measurements in representative test runs from Fig. 3.

Supplementary Discussion 6

Time traces of the Coulomb force measurement along with those of the respective variables (gas pressure or gap distance) for multiple representative test runs presented in Fig. 3 are included in Supplementary Fig. 8.

Supplementary References

1. Liu, D. et al. Standardized measurement of dielectric materials' intrinsic triboelectric charge density through the suppression of air breakdown. *Nature Communications* 13, 6019 (2022).
