# Supplementary material for: Measuring gas discharge in contact electrification
Source: Nat Commun. 2023 Dec 7;14:8100. doi: 10.1038/s41467-023-43721-1 (PMC10703932; doi:10.1038/s41467-023-43721-1)
Supplement: Supplementary file 3 — Description of Additional Supplementary Files [file 41467_2023_43721_MOESM3_ESM.pdf]

Description of Additional Supplementary files

Supplementary Movie 1 - Deflections and oscillations of load cell during surface separation and gas breakdown events.
